# The loss of SMG1 causes defects in quality control pathways in *Physcomitrella patens*

**DOI:** 10.1093/nar/gky225

**Published:** 2018-03-27

**Authors:** James P B Lloyd, Daniel Lang, Andreas D Zimmer, Barry Causier, Ralf Reski, Brendan Davies

**Affiliations:** 1 Centre for Plant Sciences, Faculty of Biological Sciences, University of Leeds, UK; 2 Plant Biotechnology, Faculty of Biology, University of Freiburg, 79104 Freiburg, Germany; 3 BIOSS - Centre for Biological Signalling Studies, University of Freiburg, 79104 Freiburg, Germany

## Abstract

Nonsense-mediated mRNA decay (NMD) is important for RNA quality control and gene regulation in eukaryotes. NMD targets aberrant transcripts for decay and also directly influences the abundance of non-aberrant transcripts. In animals, the SMG1 kinase plays an essential role in NMD by phosphorylating the core NMD factor UPF1. Despite SMG1 being ubiquitous throughout the plant kingdom, little is known about its function, probably because SMG1 is atypically absent from the genome of the model plant, *Arabidopsis thaliana*. By combining our previously established *SMG1* knockout in moss with transcriptome-wide analysis, we reveal the range of processes involving SMG1 in plants. Machine learning assisted analysis suggests that 32% of multi-isoform genes produce NMD-targeted transcripts and that splice junctions downstream of a stop codon act as the major determinant of NMD targeting. Furthermore, we suggest that SMG1 is involved in other quality control pathways, affecting DNA repair and the unfolded protein response, in addition to its role in mRNA quality control. Consistent with this, *smg1* plants have increased susceptibility to DNA damage, but increased tolerance to unfolded protein inducing agents. The potential involvement of SMG1 in RNA, DNA and protein quality control has major implications for the study of these processes in plants.

## INTRODUCTION

Eukaryotic gene expression is optimized through many regulated steps, including the differential incorporation of exonic and intronic sequences in mRNA (alternative splicing; AS). AS has the capacity to alter both the protein coding potential of a transcript and its stability. Nonsense-mediated mRNA decay (NMD) is a process that influences the steady state levels of specific transcripts by targeting them for decay (1). NMD was originally characterized as a quality control pathway that degrades transcripts with premature termination codons (PTCs), introduced by mutations or splicing errors (1). However, it is now clear that NMD plays a more substantial role, since it has been shown to influence the steady state expression of between 1% and 10% of genes in flies, worms, mammals and *Arabidopsis thaliana* (2–7). Additionally, AS-coupled to NMD (AS-NMD) is an emerging regulatory mechanism in plants and animals, in which splicing differentially includes or excludes PTCs from transcripts, thereby altering the stability of transcripts by shifting them in and out of the influence of NMD (8,9).

PTCs are distinguished from normal termination codons due to their location in ‘unusual’ contexts (10). For example, a stop codon positioned ≥50 nucleotides upstream of an splice junction can trigger decay of the transcript (11–14); this is known as the 50 nt rule (14). The presence of a long 3′ untranslated region (3′ UTR) constitutes another ‘unusual’ context, which has been reported to trigger NMD in plants, animals and fungi (12,15–17). Upstream open reading frames (uORFs) and conserved peptide uORFs can also trigger NMD (7,18,19). In mammals, it is estimated that a third of intron-containing genes could be subject to AS-NMD (20,21), and at least 13–17% of *A. thaliana* intron-containing genes undergo AS-NMD (22,23). Many of these splicing events are conserved, and are thus likely to be regulated events, required for correct expression of the targeted genes (8,24).

The RNA helicase, UPF1, is a core component of the NMD machinery. UPF1 is essential for viability in *A. thaliana*, with the null mutant allele exhibiting seedling lethality (25). Expression of AtUPF1 in tobacco leaves revealed that, as in animals, plant UPF1 is phosphorylated (26,27). However, the kinase responsible is unknown. In animals, the kinase, SMG1, phosphorylates UPF1, but *SMG1* is absent from the *A. thaliana* genome (28–30); *SMG1* is present in nearly all other examined plants, including *Arabidopsis lyrata* (29,30), making the model plant, *A. thaliana*, the exception rather than the rule (29,30). We recently showed that SMG1 functions in the NMD pathway of the moss *Physcomitrella patens* (29). The ubiquitous presence of *SMG1* in plants other than *A. thaliana* and *Capsella rubella*, and its conserved role in NMD, imply that this RNA quality control mechanism could be more similar in the plant and animal kingdoms than previously thought.

Having established moss as a model to characterize the function of SMG1 in plants, we performed RNA-seq to examine changes in gene expression and alternative splicing upon the loss of SMG1. We found that 32% of expressed multi-isoform genes produce one or more isoforms targeted to NMD, similar to the number described in mammals (20,21). A machine learning approach allowed us to identify features that are important for NMD target recognition: revealing that splice junctions in 3′ UTRs are an important determinant of NMD-sensitivity. Surprisingly, we found that not only was the NMD quality control pathway compromised, but also other quality control pathways were disrupted in *smg1* mutant plants. The unfolded protein response (UPR) was activated in *smg1* plants and, when exposed to protein unfolding drugs, mutant plants were more resistant than wild-type (WT) plants, suggesting that SMG1 may have a role in repressing the UPR. Additionally, DNA damage repair genes were also activated in *smg1* plants, which were more susceptible to DNA damage than WT plants, suggesting that SMG1 is needed for normal DNA damage repair. This highlights the importance of SMG1 in plant growth and the need to study the unfolded protein response and the response to DNA damage in plants other than *A. thaliana*.

## MATERIALS AND METHODS

### Plant growth, treatments and phenotypic examination

*Physcomitrella patens* ssp. *patens* (Hedwig) ecotype ‘Gransden 2004’ (31,32) was cultured on BCDAT medium at 25°C under continuous light (33). Lawns of protonemal filaments were grown on BCDAT medium overlaid with a cellophane disc and sub-cultured by homogenisation at seven-day intervals. To assess the reaction of moss to drug treatment, moss tissue five- to six-days post-homogenisation, was inoculated as ‘spot inocula’ on BCDAT supplemented with drug or solvent control and grown for three weeks. To induce DNA damage moss was treated with 100 or 200 ng/ml bleomycin (Euro Nippon Kayaku GmbH, Frankfurt, Germany). To induce the unfolded protein response of the endoplasmic reticulum BCDAT medium was supplemented with tunicamycin (Tm) 2.5 μg/ml or DMSO solvent control. To induce the unfolded protein response across the whole cell BCDAT medium was supplemented with 10 mM l-azetidine- 2-carboxylic acid (AZC), a proline analogue, or 10 mM proline as a control for growth. To estimate moss colony area, photographs of moss plates were taken, the image software ImageJ was used to convert the images into binary format and the number of pixels corresponding to a colony/plant measured. Moss colony size was normalised between plates and converted into mm^2^ by estimating the area of the plate (34). To test for the drug-genotype interaction in the Tm treated plants, we implemented a non-parametric version of the ANOVA test (Aligned Rank Transform test) in R (35), to account for non-normality and non-homogeneity of variances, even after log-transformation.

### RNA Isolation, RNA-seq and qRT-PCR

Total RNA was collected from 100 mg of protonema tissue, five-days post homogenisation using the RNeasy Plant Mini Kit with on-column DNase I treatment (Qiagen). RNA for RNA-seq was sent to GTAC Biotech (Constance, Germany) for library preparation using the TruSeq sample preparation kit (Illumina) and sequencing on the Illumina HiSeq2000 to generate single-end 100 nt long reads. Two biological replicates were collected for WT and each of the two mutant lines. Template for quantitative (q)RT-PCR was made using 1 μg of total RNA and iScript cDNA synthesis Kit (Bio-Rad). qRT-PCR was carried out using a Bio-rad CFX96 Real-Time System with SsoFast EvaGreen Supermix (Bio-Rad). To monitor the expression of unfolded protein responsive genes, moss tissue five-days post-homogenisation was inoculated as ‘spot inocula’ on BCDAT supplemented with 1 μg/ml of tunicamycin or DMSO as solvent control and grown for two weeks before tissue was collected for RNA extraction. All primers are listed in Supplemental Table S1.

### Transcript assembly, isoform selection and differential analysis of expression and splicing

The reads generated by RNA-seq were mapped to the genome using TopHat (tophat-1.3.1.Linux_x86_64 custom version supporting additional bowtie1 parameters), which can map reads spanning exon-exon junctions (36) employing the parameter configuration: -I 20000 (maximum intron size), –segment-mismatches 2, –segment-length 18, –microexon-search, –maqerr 90, –bowtie-strata, –bowtie-all, –bowtie-best, –max-multihits 50 and –initial-read-mismatch 3, and using the version 1.6 of the moss genome as a reference (37). Transcript- and gene-wise read counts were obtained using htseq-count (38). Differential gene expression analysis was carried out using edgeR (39), DESeq (40) and NOISeq (41) with a significance threshold of corrected *P* < 0.05. Genes were considered differentially expressed if they were identified by at least two of the three tools in both *smg1* mutants. GO and MapMan enrichments were then performed on the differentially expressed genes using custom R scripts utilizing the weight algorithm of the topGO package for GO analysis (for scripts, see Zenodo archive: https://doi.org/10.5281/zenodo.826164).

To identify novel transcripts, mapped reads of the individual libraries were assembled into candidate transcripts using Cufflinks (42), which were subsequently clustered and combined together with the V1.6 gene structures into consensus gene models using PASA and a set of custom perl scripts (for scripts, see Zenodo archive: https://doi.org/10.5281/zenodo.826164). The main open reading frame (ORF) was chosen based on homology, protein length and isoform expression. Using the improved annotation, we counted reads in each library which were specific to individual splicing events and used these event-specific read counts (Perl script; https://doi.org/10.5281/zenodo.826164) to perform differential expression analysis at the splice site level using the general linear model method implemented in edgeR (39). Transcript expression was also measured, with htseq-count (38). We contrasted isoform abundances in mutant and wild-type lines by estimating CPM-abundance, log-Fold change and FDR-corrected *P*-value for each splicing event and whole isoform separately. The FDR-corrected *P*-value threshold was *P* < 0.01. Change of protein structure/function/domains was evaluated considering the BLAST homology and PFAM domain coverage of the reference and the alternative transcripts. For this we compared alignments of both domain and BLAST matches. If these were altered (difference in length or number) then we considered the protein to be significantly altered.

### Identification of NMD targeting features and prediction of NMD targets using ensemble (Machine) learning

In order to utilize the classified splice events to identify common NMD targeting features, and to discriminate between true NMD targets and secondarily deregulated isoforms in the *smg1* mutants, we collected 361 additional structural and sequence features in addition to the expression data described above. First, the categorical values were split up into binary features (e.g. AS-type.alt_acceptor = 1|0). In addition, we collected numeric attributes describing the expression level in the mutants/WT, measuring 46 features of gene structure and uORFs, 207 sequence compositional features of transcript- and event-level contexts, three features describing cytosine methylation levels (43) at splice sites as well as 67 frequencies of overrepresented motif and K-mers. Subsequently we compacted the feature vector by filtering near-zero variance and reducing correlated (>75%) features using the caret package (44). Two versions of the machine learning approach were performed: The first used all available transcript attributes, including the expression data (EXP), while the second used only structural and sequence features, not features related to expression data (NOEXP).

A training set of 107 transcripts which were either likely NMD targets or non-NMD targets were selected (Supplemental Table S2). NMD targets were chosen by reviewing many loci in the Genome Browser by identifying alternative isoforms which led to increases in expression upon NMD inhibition and introduced an early stop codon. Non-NMD targets were chosen due to the lack of change in expression in *smg1* plants. The training set was established in an iterative procedure that involved multiple rounds of manual curation, training, prediction, repeat. We selected a random sample of targets for manual curation. Random sampling was not only carried out on the entire data set as a whole, but on subsets that were defined based on exploratory analysis of features not necessarily linked to NMD, like e.g. alternative donors or CDS changing splicing events, to get a broad representation of the data. We performed cross-validation to ensure that we did not over-fit. Additionally, some of the training set was withheld from rounds of machine learning, and then tested on the excluded training set list. This ensured that some features were not being over-predicted before we proceeded to the final round where we used the whole training set to generate the final, cross-validated model. With this training set, we utilized the resulting feature vector for each of the identified splicing events to perform supervised classification following an ensemble learning/bagging approach that utilized 76 distinct machine learning methods. Each of the 76 compatible, classifiers implemented in the caret package (44) was trained and evaluated using three times repeated 5-fold cross-validation. The trained models were filtered for models with Accuracy ≥95% (EXP) and ≥75% (NOEXP) in the cross-validation procedure. The resulting 19 (EXP) and 11(NOEXP) models were used for predicting the whole dataset. Each model results in an event classification as: is.NMD = true or false.

## RESULTS

### Analysis of differential gene expression in *smg1* moss

**Figure 1.**
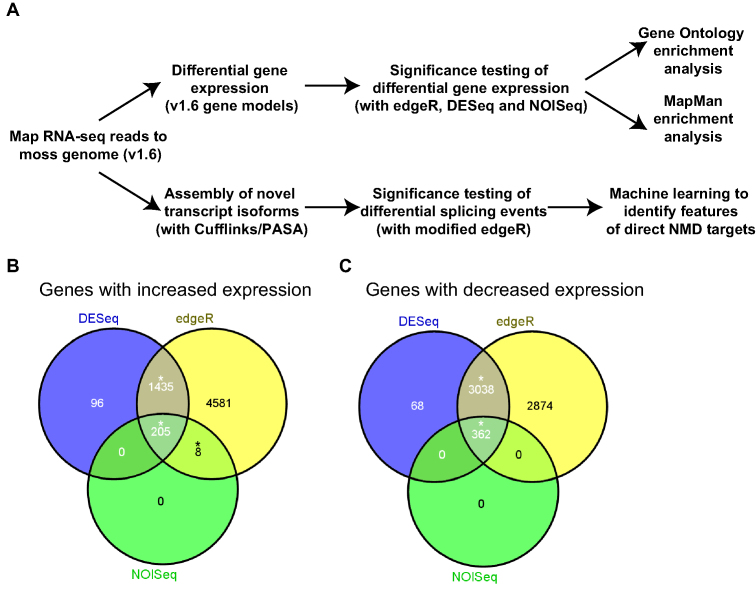
Analysis of differential expression from *smg1* mutant plants. (**A**) Outline of the analysis pipeline to find differential gene expression and differential alternative splicing in the *smg1* mutant plants. (**B** and **C**) Genes up-regulated and down-regulated, respectively, in *smg1*Δ lines when compared to WT (*P* < 0.05). Three different tools were used to assess if a transcript was up- or down-regulated (DESeq, edgeR and NOISeq). During the first round of selection, only genes that were differentially regulated in at least two tools were taken forward (overlap is indicated with an asterisk).

To investigate the influence of SMG1 on gene expression, we analysed the transcriptomic changes that occur upon the loss of *SMG1* in the moss *P. patens*. RNA was collected from WT moss and two independently generated deletion mutants (*smg1-1* and *smg1-2*) (29) and subjected to high-throughput RNA-sequencing (RNA-seq). RNA-seq reads were mapped to the moss genome, with an average mapping rate of 89% (Supplemental Table S3). The subsequent analysis followed a two-pronged approach (Figure 1A). First, we identified differentially expressed genes using the published version 1.6 gene models of the moss genome (37). Second, we inferred isoforms via transcript assemblies, examined differential splicing at the event level and then took a machine learning approach to identify the transcript features that target transcript isoforms to NMD (Figure 1A).

### Identifying dysregulated pathways in *smg1* mutant plants

We generated a conservative list of differentially expressed genes by using three computational tools: DESeq, edgeR and NOISeq (40,41,45). Genes that showed significantly changed expression in both mutants (*smg1-1* and *smg1-2*), by at least two tools, were considered to be differential (Supplemental Table S4). Using these strict criteria, we identified 1648 and 3400 genes with increased and decreased steady state expression in *smg1*, respectively (Figure 1B and C). Therefore, large-scale changes in moss gene expression result from the loss of *SMG1*, with 13% of the moss genes showing differential expression. This is consistent with the wide-ranging transcriptional changes observed in NMD compromised flies, worms, human cells and *A. thaliana* (4,46–48).

Given the previously identified *smg1* mutant phenotype of reduced gametophore number (29), we examined the expression of genes associated with the early development of gametophores (buds) to see if they were reduced in *smg1* plants. Genes with reduced expression in *smg1* plants show a significant enrichment for previously identified bud induced genes (49) (Hypergeometric test: *P* = 1.14 × 10^−103^), including five known bud marker genes (50) (Table 1). These data demonstrate that our RNA-seq analysis can capture the known phenotypic consequences of the deletion of *SMG1*.

**Table 1.** Genes involved in bud development have lower expression in *smg1* mutant moss

Gene ID	Gene name	Fold change (log_2_) in *smg1* plants	Significant tests
Pp1s25_23V6	BIP1	–188.22	2
Pp1s400_1V6	BIP2	–152.61	2
Pp1s31_134V6	BIP3	–69.08	2
Pp1s190_39V6	BIP4	–37.43	3
Pp1s8_135V6	BIP6	–34.66	2

The significant tests column reflects the number of differential testing tools (DESeq, edgeR and NOISeq) in which the gene was significantly changed.

To discover other biological processes that are disrupted after *SMG1* deletion in moss, the conservative list of differentially expressed genes was used for gene ontology (GO) and MapMan (51) enrichment analysis. Amongst the genes that showed enhanced expression in *smg1* mutants, the most highly enriched MapMan term is DNA synthesis/chromatin structure (Table 2). Manual inspection of these genes revealed that at least four of them have a role in DNA repair. DNA repair and DNA recombination were also enriched terms in the GO analysis (Supplemental Table S5), indicating an over-expression of genes relating to DNA damage repair in the *smg1* mutants. A dysregulation of the DNA repair pathway upon the loss of *SMG1* is reminiscent of a defect seen in animal cells with knocked down *SMG1* (52). The second most highly enriched MapMan term associated with genes with an increased steady state expression in *smg1* plants was stress/abiotic/heat, which completely overlapped with another MapMan term, chaperones and co-chaperones/HSP70s/chaperones (Table 2 and Supplemental Table S6). The majority of these genes (21/23) encode predicted chaperones, co-chaperones or proteases with predicted roles in the degradation of unfolded proteins (Supplemental Table S7). These genes appear to be associated with various cellular compartments including the cytosol, chloroplast and mitochondria. The unfolded protein response (UPR) of the endoplasmic reticulum (UPR^ER^) has been the focus of many studies in animals and plants (53,54). A manual search for moss homologues of genes implicated in the UPR^ER^ in *A. thaliana* revealed that expression of genes such as *BiP* and *Derlin-1* is increased in *smg1* (Supplemental Table S7). Genes showing reduced expression in the *smg1* mutants were also identified and categorized by terms such as photosynthesis, translation, cell wall modification and leucine rich repeat receptor kinases, suggesting a decrease in normal growth and metabolism in the absence of *SMG1* (Supplemental Tables S5 and S6). Taken together, analysis of differential gene expression indicates that the loss of *SMG1* leads to the dysregulation of many genes, with stress response genes relating to DNA damage and the unfolded protein response being over-expressed; the biological processes enriched among the down-regulated genes are consistent with an overall developmental retardation, hindered stress-response, and metabolic impairment.

**Table 2.** Over-represented MapMan terms of genes with increased steady state expression in *smg1* plants

MapMan term description	*P* value
DNA synthesis/chromatin structure	4.58E–03
Stress abiotic heat	4.66E–03
Transport ABC transporters and multidrug resistance systems	1.48E–02
Chaperones and cochaperones HSP70s chaperones	2.33E–02
RNA processing splicing	2.60E–02

### The loss of *SMG1* leads to disrupted DNA and protein quality control systems

**Figure 2.**
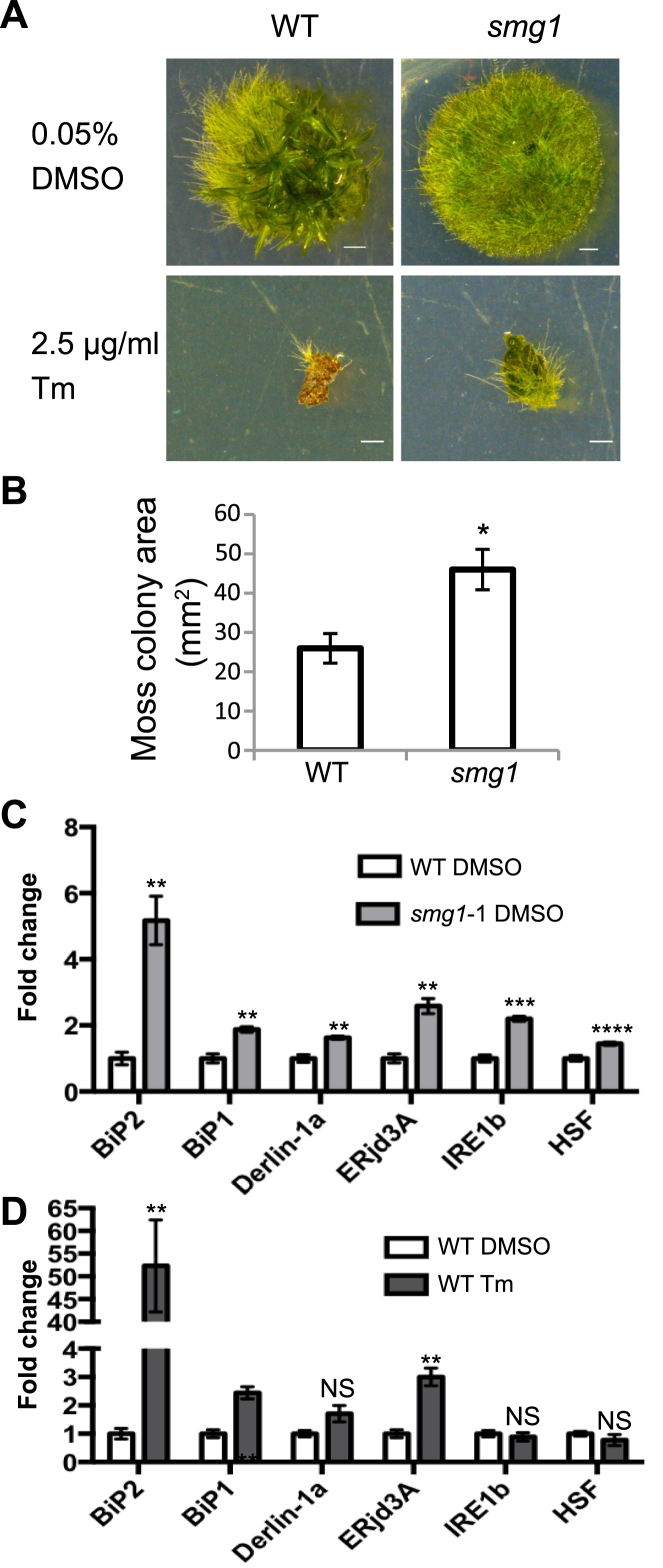
*smg1* plants are partially resistant to the unfolded protein inducing drug tunicamycin (Tm). (**A**) Three week old plants grown on Tm or solvent control (DMSO). Scale bar is 1 mm. (**B**) Moss colony size on Tm (2.5 μg/ml). *n* = 5–12. Asterisks represent a statistically significant difference from WT using an unpaired *t* test *P* < 0.05. A significant drug/genotype interaction was identified (Aligned Rank Transform test; *P* = 4.14 × 10^−5^), along with a significant drug treatment (Aligned Rank Transform test; *P* = 7.25 × 10^−11^) and genotype (Aligned Rank Transform test; *P* = 7.02 × 10^−5^) effect. (**C**) qRT-PCR analysis of *BiP1* (Pp1s181_3V6), *BiP2* (Pp1s288_23V6), *Derlin-1a* (Pp1s213_66V6), *ERjd3A* (Pp1s368_19V6), *IRE1b* (Pp1s34_189V6) and *HSF* (Pp1s31_388V6), expression in WT and *smg1*Δ line 1 (DMSO treated as solvent control). (**D**) qRT-PCR analysis of *BiP1* (Pp1s181_3V6), *BiP2* (Pp1s288_23V6), *Derlin-1a* (Pp1s213_66V6), *ERjd3A* (Pp1s368_19V6), *IRE1b* (Pp1s34_189V6), and *HSF* (Pp1s31_388V6), expression in WT treated with Tm (1 μg/ml) or untreated (DMSO solvent control). The fold change indicates the amount of target expression normalized to that of *PpEF1α* and relative to WT levels. Error bars represent the standard error of the mean from three biological replicates. Asterisks indicate conditions with a statistically significant difference from WT (DMSO solvent control) using an unpaired *t* test (*P < 0.05; **P < 0.01; ***P < 0.001; ****P < 0.0001). WT DMSO control in panel C and D represent the same data.

Many genes involved in the UPR showed increased steady state expression in *smg1* plants compared to WT (Table 2), suggesting that *SMG1* is important for the normal response to unfolded proteins. We treated moss with tunicamycin (Tm) to induce unfolded proteins in the ER (55–59) and found that *smg1* lines grown on Tm were larger and greener than WT controls (Figure 2A and B). To confirm that this is not a drug-specific effect, we also treated WT and *smg1* lines with the proline analogue, L-azetidine-2-carboxylic acid (AZC), previously shown to induce unfolded proteins in *A. thaliana* (57,59). As with Tm, *smg1* plants grown on AZC were larger and greener than WT controls (Supplemental Figure S1). *smg1* colonies are typically larger than WT colonies on control media, except when supplemented with proline as a control for AZC (Supplemental Figure S1A). However, we still see *smg1* plants outgrowing WT plants on AZC, further supporting our conclusion that *smg1* plants are better able to grow under high unfolded protein stress than WT plants. We then selected six UPR-related genes for which the RNA-seq analysis showed stronger expression in the *smg1* mutants than in the WT, for validation using qRT-PCR. All six genes showed elevated expression in *smg1* mutant lines (Figure 2C), in agreement with our RNA-seq analysis. We found that some of these genes (*BiP1, BiP2* and *ERjd3A*) were also elevated in moss treated with Tm (Figure 2D). *BiP2* in particular is highly inducible by Tm treatment, with steady state levels increasing over 50-fold. Taken together, these data show that *smg1* mutant lines exhibit partial resistance to inducers of unfolded proteins, which may be due to an increased synthesis of chaperones and other proteins involved in clearing the cell of unfolded proteins.

**Figure 3.**
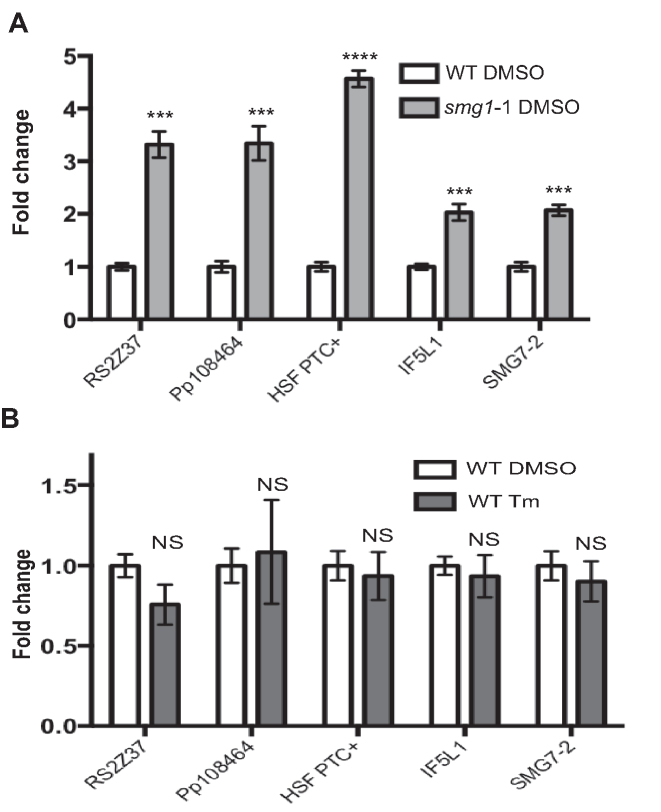
NMD targets are not increased in Tm exposed plants. (**A**) The expression of NMD targets *RS2Z37* (Pp1s69_23V6), *Pp108464* (Pp1s270_54V6), *HSF* Pp1s31_388V6*eIF5L1* (Pp1s626_4V6), and *SMG7-2* (Pp1s311_73V6) in the *smg1* mutant plants treated with DMSO as a solvent control. (**B**) The expression of NMD targets *RS2Z37* (Pp1s69_23V6), *Pp108464* (Pp1s270_54V6), *HSF* (Pp1s31_388V6), *eIF5L1* (Pp1s626_4V6), and *SMG7-2* (Pp1s311_73V6) in WT plants exposed to two weeks of Tm (1 μg/ml). WT DMSO control in panel A and B represent the same data. The fold change indicates the amount of target expression normalized to that of *PpEF1α* and relative to WT levels. Error bars represent the standard error of the mean from three biological replicates. Asterisks indicate conditions with a statistically significant difference from WT (DMSO solvent control) using an unpaired *t* test (*P < 0.05; **P < 0.01; ***P < 0.001; ****P < 0.0001).

The activation of UPR^ER^ and some other cellular stresses in animal cells are known to inhibit NMD (19,60–62). Therefore, we tested the expression of several NMD targets in Tm treated moss to determine whether the UPR^ER^ also reduces the activity of NMD in moss. Five transcripts were tested, four of which have previously been shown to be NMD targets in moss (29), and one novel PTC-containing splice variant (HSF PTC+) identified in this study. As expected, all these transcripts are increased in the *smg1* mutant (Figure 3A), but Tm treatment does not significantly elevate their expression (Figure 3B). Our data suggest that activation of the UPR does not suppress the NMD pathway in moss, as it does in animals (19,60–62).

**Figure 4.**
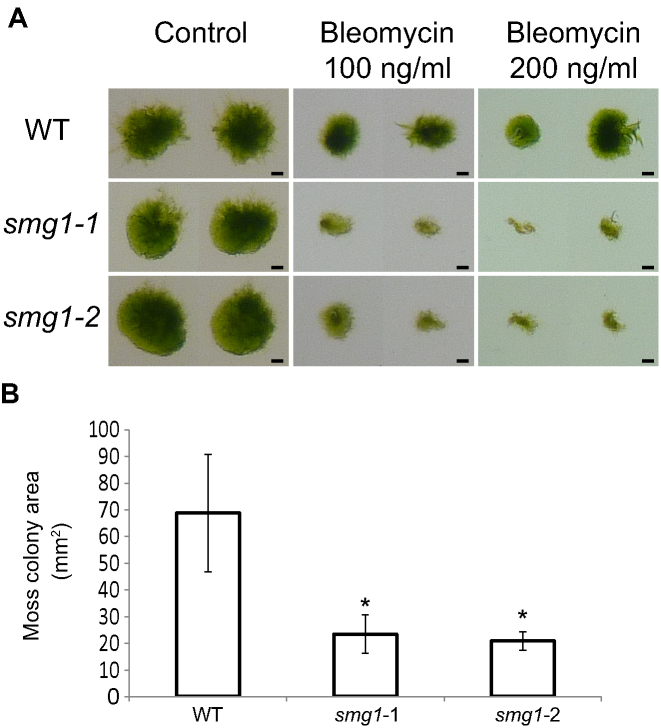
*smg1* lines are more susceptible than WT to bleomycin. (**A**) Two week old moss plants grown on media with or without bleomycin. Two representative colonies of each line are shown for each treatment. Scale bar is 1 mm. (**B**) Moss colony size after three weeks growth on 100 ng/ml bleomycin. Data is the mean from 6–12 plants. Error bars represent standard deviation. Asterisks indicate lines with a statistically significant difference from the WT using an unpaired *t* test *P* < 0.05.

Our differential gene expression analysis also showed that many genes with an increased steady state expression in moss *smg1* mutant plants were associated with DNA repair (Table 2, Supplemental Tables S5 and S6). In mammals, SMG1 has an NMD-independent role in the DNA repair pathway and the loss of *SMG1* increases the expression of DNA damage markers and DNA damage susceptibility (52,63). Therefore, moss was grown on the DNA damage inducing agent bleomycin, which has previously been used to test the susceptibility of moss mutants to DNA damage (34,64,65). After three weeks of growth on bleomycin, *smg1* plants were significantly smaller and less green than WT plants (Figure 4), suggesting that the loss of *SMG1* leads to an increased susceptibility to DNA damage, implicating SMG1 in the moss DNA repair pathway.

Taken together, these data show that the loss of SMG1 leads to the dysregulation of multiple quality control pathways in moss plants, evident in gene expression changes as well as the altered responses to drugs that induce either unfolded protein, or DNA damage. Therefore, SMG1 could be an important factor for quality control at the DNA and protein level, in addition to its established role in RNA quality control.

### NMD in moss is triggered by the presence of 3′ UTR splice junctions

**Figure 5.**
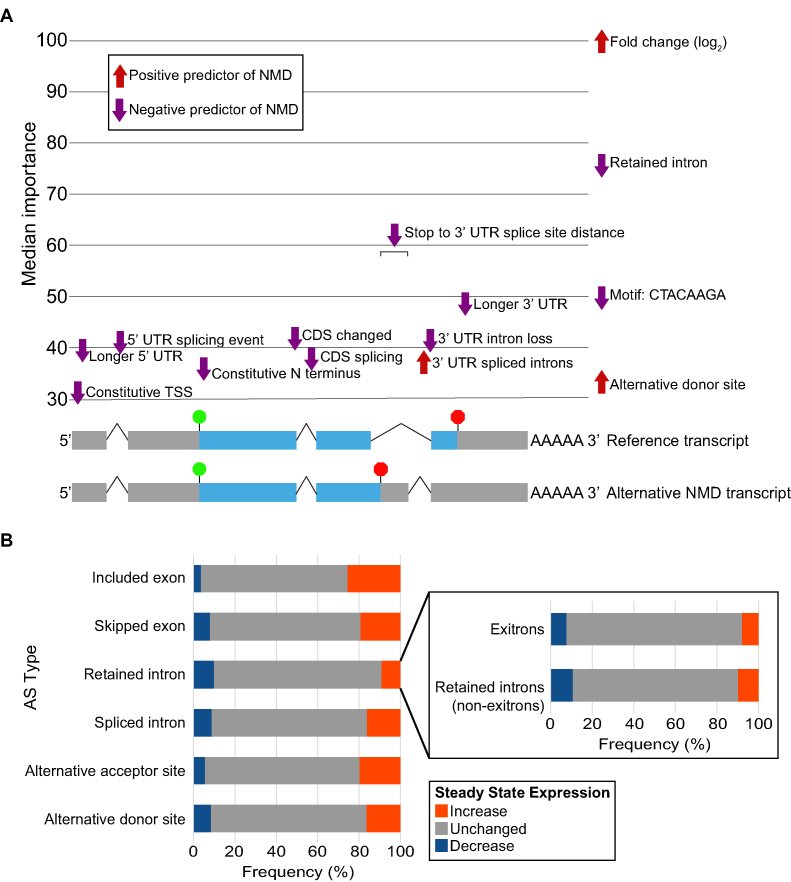
Transcript attributes influencing NMD target identification. (**A**) Factors that influenced NMD target status. The plot quantifies how well transcript attributes distinguish between NMD-targeted and non-NMD targeted events/transcripts in the ensemble machine learning approach. Transcript attributes act as either positive (red) or negative (purple) predictors of NMD-targeted status. Transcript attributes are located above the relevant feature of the example transcript models (bottom), with the exception of transcript-wide features (fold change (log_2_), retained intron, motif: CTACAAGA and alterative donor site), located on the right of the plot. Example transcript models represent two transcript isoforms, one non-NMD target (top) and one NMD target (bottom). Transcript attributes are ranked by ‘median importance’: Importance is the relative predictive power of each transcript attribute in identifying NMD-targeted events/transcripts. The median importance is taken from across the various machine learning tools used in this study. Abbreviations used in plot: TSS is transcriptional start site, CDS is coding sequence, and UTR is untranslated region. (**B**) The frequency of different AS types with increased, decreased or unchanged alternative splicing after the loss of *SMG1*. The AS types included: Included exon (retained exon; *n* = 2260), skipped exon (*n* = 829), retained intron (*n* = 58 459), spliced intron (*n* = 4636), alternative acceptor site (n = 5106), and alternative donor site (*n* = 4110). The frequency of exitrons (*n* = 18901) and other retained introns (*n* = 39 558) after loss of *SMG1*. Exitrons are defined as retained introns that are within a coding region and do not introduce a PTC. More differential exitrons have increased than differential retained introns (Fisher's exact test one-tailed test, *P* = 0.0056).

Various transcript features, including long 3′ UTRs and 3′ UTR splice junctions, have been proposed to mark a stop codon as aberrant, triggering NMD in various organisms, but it is currently unclear what features determine whether a transcript is an NMD target in moss. Using the RNA-seq data from *smg1* plants and a machine learning approach, we were able to identify many features linked to the NMD-sensitivity of a transcript. First, we used Cufflinks (42) and PASA (66) to infer transcript isoforms from RNA-seq reads and existing transcripts, and to classify AS types in the newly assembled transcriptome. Then reads mapped unambiguously to the splicing events identified by PASA were tested for differential abundance in *smg1* plants against WT plants using the generalized linear modelling in edgeR (45). This allowed us to identify direct targets of AS-NMD. A list of 108 manually curated NMD-sensitive and NMD-insensitive transcript events (Supplemental Table S2) was used as a training set for cross-validated training of 76 distinct machine learning techniques, available via the caret package (44). To identify the transcripts that trigger NMD, 328 transcript attributes were used by the machine learning approach, including details of transcript expression, splicing, and the presence of motifs. We identified 14128 events as NMD-targeted, representing 32% of annotated genes with expressed multiple isoforms. The transcript attributes most useful in determining NMD-sensitivity are presented in Figure 5A and Supplemental Figure S2. Some of these transcript attributes reveal mechanistic insights into how NMD targets are identified in moss. The transcript attribute ‘3′ UTR spliced introns’ is a positive predictor of a transcript targeted to NMD (Figure 5A), indicating that moss recognizes premature stop codons by the presence of a downstream exon-junction complex, as do animals and other plants (12,14,17,23,67). Surprisingly, we found that long 3′ UTRs are not positive predictors of NMD-targeting (Figure 5A), as has been suggested for animals and other plants (12,15,17). We also found that the more distant a 3′ UTR splice junction was from the stop codon, the less efficient at triggering NMD it appeared to be (Figure 5A). Other features include an under-representation of specific codons in uORFs (ATC, AAC, and GGT) and/or an over-representation (CAGTTGAAATTT and GTGAAAVTTTTC) or under-representation (CCAACATCAT, CTTGGCTA, and THTCAWGGGT) of certain motifs in the NMD targeted transcripts (Figure 5A and Supplemental Figure S2). However, it is unclear how these attributes alter NMD-sensitivity.

The GO enrichment of predicted direct AS-NMD targets (Supplemental Figure S3) reveals that splicing coupled to NMD is important for controlling the expression of a wide range of genes with diverse functions. Therefore, the mechanism of AS-NMD has a broad role in gene expression and is not limited to controlling a single process. We found that nearly all (96%) splicing introduced PTCs disrupt conserved domains of the encoded protein (Supplemental Figure S4), highlighting the importance of NMD in maintaining the proteome and the need for correct identification of NMD targeted transcript isoforms when curating a computationally generated transcriptome. Taken together, these data suggest that the NMD pathway of moss targets transcripts to NMD predominately via the presence of a 3′ UTR located splice junction but not a long 3′ UTR.

Transcript attributes related to the type of alternative splicing have predictive power in determining whether a transcript is targeted to NMD (Figure 5A). The presence of an alternative donor site is a positive predictor of NMD targeting, while the presence of a retained intron acts as a negative predictor (Figure 5A). Indeed, the steady state expression of transcripts with alternative donor sites tend to increase rather than decrease in the *smg1* plants (Figure 5B). This pattern is also seen for transcripts with included exons, skipped exons, spliced introns, and alternative acceptor sites, but not for retained introns (Figure 5B). A similar fraction of retained introns increases and decreases after NMD is inhibited (Figure 5B), suggesting that most changes in transcript with retained introns are linked to secondary changes rather directly leading to NMD. Exitrons are a set of coding retained introns that do not introduce PTCs (68), we found that exitrons and other retained introns has a similar pattern of change after NMD was inhibited (Figure 5B), further evidence that retained introns are not used as a global mechanism to induce NMD. Therefore, not all AS types are equally used to target transcripts to NMD.

**Figure 6.**
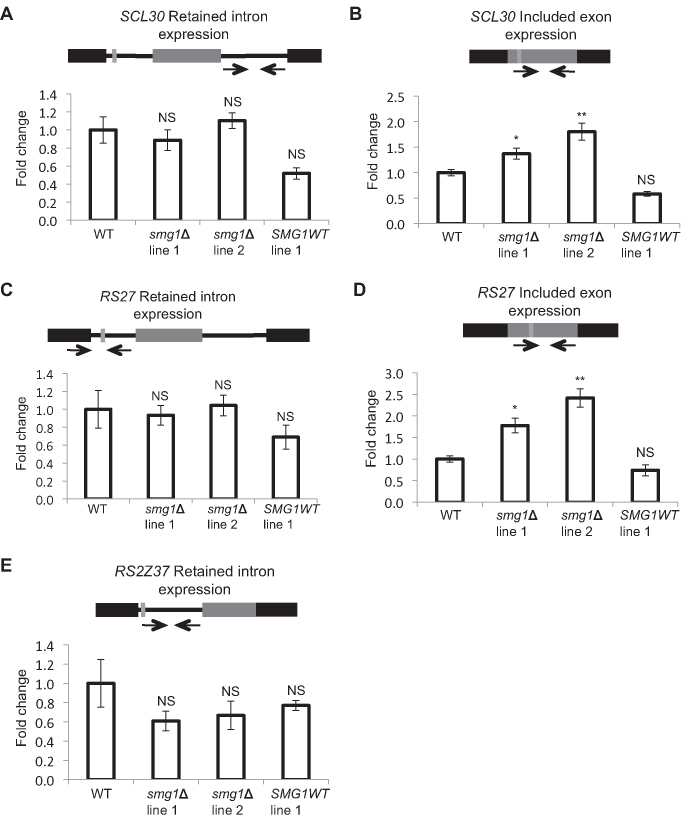
Isoforms with PTCs introduced by a retained intron do not have increased steady state expression in *smg1* mutants. (**A**) qRT-PCR analysis of the *PpSCL30* (Pp1s183_39V6) retained intron PTC+ variant. (**B**) qRT-PCR analysis of the *PpSCL30* (Pp1s183_39V6) included exon PTC+ variant. (**C**) qRT-PCR analysis of the *PpRS27* (Pp1s173_12V6) retained intron PTC+ variant. (**D**) qRT-PCR analysis of the *PpRS27* (Pp1s173_12V6) included exon PTC+ variant. (**E**) qRT-PCR analysis of the *PpRS2Z37* (Pp1s69_23V6) retained intron PTC+ variant. (**A–E**) The fold change indicates the amount of target expression normalized to that of *PpEF1α* and relative to WT levels. Error bars represent the standard error of the mean from three biological replicates. Asterisks indicate conditions with a statistically significant difference from WT using an unpaired *t* test (*P < 0.05; **P < 0.01; ***P < 0.001; ****P < 0.0001). NS, not significantly different. The black boxes represent constitutive exons, grey boxes represent alternative exons and black lines represent unspliced introns. Light grey lines represent introduced PTCs. Change in expression *PpRS2Z37* (Pp1s69_23V6) alternative acceptor site PTC+ variant was already reported (29).

Retained intron transcripts have been reported as NMD targets in various eukaryotes (48,69,70). However, we (Figure 5) and others (22,23,71) have found many retained intron transcripts to be resistant to NMD. To confirm this result, we selected three retained intron events to examine in more detail. In addition to having retained intron isoforms, *PpSCL30, PpRS27* and *PpRS2Z37*, all have NMD targeted isoforms resulting from an included exon (*PpSCL30* and *PpRS27*) or alternative acceptor site (*PpRS2Z37*). qRT-PCR revealed that the predicted NMD targeted isoforms resulting from included exons (Figure 6) or alternative acceptor sites (29) are present in increased abundance in the *smg1* mutant lines, but the retained intron isoforms show no such increase (Figure 6), supporting the notion that some retained intron transcripts are resistant to NMD in moss, as has been found for *A. thaliana* and mammals (22,23,71).

## DISCUSSION

We have previously discovered that SMG1 is widespread in the plant kingdom and functions in the NMD pathway of the moss *Physcomitrella patens* (29). The specific loss of *SMG1* in the *A. thaliana* makes it impossible to study the role of this NMD factor in this widely used plant model system. We therefore took advantage of moss *smg1* mutants to examine the functional role of SMG1 in plants at the transcriptomic level. We show that the NMD factor SMG1 has an important role in moss growth and development and that the loss of *SMG1* leads to widespread changes in the steady state expression of thousands of genes and splice variants. As in animals and *A. thaliana*, we find that AS-NMD is a major mechanism of gene regulation, influencing up to 32% of genes with multiple isoforms; we estimate that between 3–18% of all splice events in moss lead to NMD. This suggests that NMD is a frequent consequence of alternative splicing and plays an important role in moss gene regulation.

Many PTC-containing transcript isoforms with retained introns are not NMD targets, adding to the evidence from other systems that retained intron transcript isoforms are NMD-resistant (22,23,71). Finally, we showed that the loss of *SMG1* affects the plant's ability to respond to both DNA damage and unfolded protein inducing drugs, suggesting an important role for SMG1 in stress responses of plants.

### Loss of SMG1 leads to dysregulation of quality control pathways in moss

SMG1 has an important role in NMD (29). Using differential gene expression analysis, we identified multiple pathways that were dysregulated in *smg1* plants, which revealed the surprising result that, in addition to this RNA quality control pathway, genes in the DNA quality control pathway (DNA repair) and protein quality control pathway (unfolded protein response) were also up-regulated (Table 2 and Supplemental Tables S5 and S6). We used a conservative approach to identify differentially expressed genes, looking only at genes reported as significant from at least two of edgeR, DESeq and NOISeq. Given the large overlap between NOISeq and the other tools, we speculate that NOISeq is the most accurate, but also the most conservative of the tools used. Not only did we find many changes in other quality control pathways of *smg1* plants, but we found that these gene expression changes translated into an altered response of *smg1* plants to both DNA damage and unfolded proteins. When compared with WT plants, *smg1* mutants show increased susceptibility to DNA damage (Figure 4) and enhanced resistance to unfolded proteins (Figure 2 and Supplemental Figure S1). This is in contrast to *A. thaliana*, where inhibition of NMD leads to an auto-immune like state (7,72), due to the increased expression of toll-like leucine-rich repeat receptors (73). In moss, we see the opposite trend, with leucine-rich repeat receptors being enriched among genes with decreased expression (Supplemental Table S6). Additionally, we do not see evidence for an auto-immune like response in moss, with no enrichment of pathogen response genes increasing in *smg1* plants (Supplemental Tables S5 and S6).

**Figure 7.**
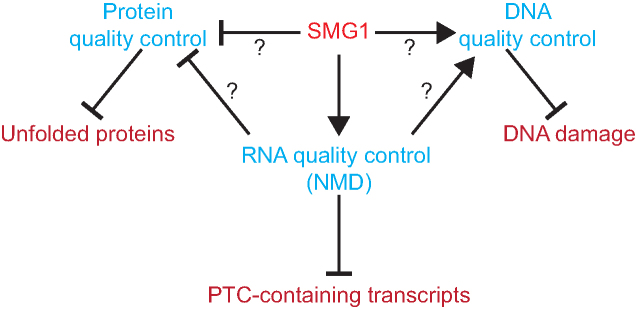
SMG1 is important for multiple quality control pathways in moss. SMG1 activates the NMD pathway, which leads to repression of PTC-containing transcripts. We suggest that SMG1, potentially acting via NMD, represses the unfolded protein response, and activates the DNA repair machinery.

It is unclear whether the activation of these pathways results indirectly from the decrease in NMD, due to the loss of a central NMD factor (SMG1), causing dysregulation of regulators normally targeted by NMD, or whether it is a direct result of SMG1 having NMD-independent functions in these pathways (Figure 7). Regarding the UPR, it is possible that the loss of NMD increases the level of unfolded proteins, for example through increased expression of truncated proteins, which could prime the mutant plants. This might allow the *smg1* plants to tolerate additional unfolded proteins. Alternatively, NMD could act as a regulatory mechanism to repress the UPR in moss (Figure 7). Future work will help to distinguish between these two possibilities. Currently, *smg1* mutants are the only known viable NMD factor knockout line. Attempts to knockout other NMD factors have been unsuccessful. For example, when we attempted to knock out the only other single-copy core NMD factor in the moss genome, *UPF2* (29), 15 independent knockout lines were obtained, however, all 15 retained a second copy of *UPF2*, suggesting lethality when removing *UPF2* from regenerating protoplasts. Our attempts to generate knockout lines for the two *UPF1* homologues have produced similar results.

In mammals, NMD targets transcription factors involved in the activation of the UPR^ER^ through the uORFs in their 5′ UTRs (19,60,62). In response to unfolded protein stress, NMD is repressed, thus increasing the expression levels of these transcription factors (60). The repression of NMD is achieved through phosphorylation of eIF2-α (60), although how this achieves NMD repression is unclear. In this study, we found no evidence for a global repression of NMD when plants are exposed to inducers of unfolded proteins (Figure 3), suggesting that if NMD regulates the UPR in plants, it operates through a different mechanism than in mammals.

Given the large number of pathways that NMD regulates (3,7), it is possible that SMG1 acts through NMD to prevent DNA damage by activating DNA repair; the compromised NMD pathway in the *smg1* mutant could conceivably result in spontaneous DNA damage to accumulating, due to the lack of repair, leading to the increased expression of DNA damage related genes observed (Table 1). *smg1* was recently identified in an unbiased screen for activators of DNA repair in *C. elegans* (74). It was then shown that mutations in other NMD factors also lead to compromised double strand break repair in *C. elegans* (74), suggesting that the NMD pathway acts to activate DNA repair, similar to what we have observed in moss (Figure 7). We found some DNA repair factors were alternatively spliced and then targeted to NMD (Supplemental Figure S3), although this is unlikely to be the cause of the changes in DNA damage response seen in the *smg1* mutant plants: NMD is acting here to degrade the PTC-containing, non-functional transcript isoform, rather than directly altering the protein-producing isoform. However, it is possible that when NMD is inhibited, truncated proteins may be translated and disrupt the activity of the full-length proteins. Alternatively to DNA repair activity being controlled via NMD, SMG1 could act directly in the DNA repair pathway (Figure 7). Such a role has been observed in humans, where SMG1 phosphorylates p53 in response to DNA damage (52,63,75), and the loss of *SMG1* leads to spontaneous DNA damage and increased susceptibility to ionizing radiation (52,63). Since plants lack a homologue of p53, SMG1 might phosphorylate a plant equivalent of p53. SOG1 has previously been proposed to be such an equivalent of p53 in plants (76–78). SOG1 is phosphorylated at SQ dipeptides by the ATM kinase, as is p53 in the mammalian DNA repair pathway (75,79). It is also known that the SMG1 kinase has a specificity for SQ dipeptides (80). It is therefore tempting to speculate that in plants SMG1 phosphorylates SOG1 at SQ dipeptides, as does ATM. SMG1 may also act directly in the regulation of splicing of important regulators of DNA repair, as has recently been demonstrated in animal cells (81). The sensitivity to bleomycin of *smg1* plants is similar to that of the recently characterised deletion of the moss *RecQ4* (65), raises the possibly of a mechanistic link. Especially as some RecQ helicases are phosphorylated by PIK kinases (82). Given the significance of DNA repair, it will be important to investigate further the mechanism through which SMG1 confers tolerance to DNA damage in moss and potentially most other plants. This is not possible with *A. thaliana* given the genome is lacking an SMG1 orthologue (28–30), highlighting the need for alternative model plants like *P. patens* that allow the study of the link between DNA damage repair and NMD.

### Downstream splice junctions are an important determinant of NMD in moss

Several features have been suggested to target transcripts to NMD in diverse organisms. These include long 3′ UTRs and 3′ UTR located splice junctions at least 50 nucleotides downstream of the stop codon. Long 3′ UTRs are proposed to act as NMD targeting features either via stalling of termination due to a greater distance between the stop codon and the poly-A binding protein or by long 3′ UTRs binding more UPF1 until a critical level is reached and NMD is activated (83). A splicing event in the 3′ UTR may deposit an exon-junction complex (EJC), which can recruit NMD factors to stimulate NMD during termination of translation at the upstream stop codon, although 3′ UTR splice junctions have been reported to trigger NMD in *Tetrahymena thermophila* without the involvement of the EJC (84). Additionally, fission yeast NMD is enhanced by splicing but is also independent of the EJC (85), suggesting that many organisms are able to sense splicing linked PTCs without involvement of the EJC. We found, among our high confidence NMD targeted isoforms, that a splice junction in the 3′ UTR was a powerful predictor of NMD, but this was not the case for long 3′ UTRs (Figure 5A). While average 3′ UTR lengths do vary between plant species, moss appears to be very typical in this regard (37), suggesting this might not be some quirk of moss. Some studies report that the average 3′ UTR length of NMD targets is greater than non-NMD targets, but this is likely due to these studies not accounting for the presence of 3′ UTR splice junctions. In human cancers, an unbiased machine learning approach identified 3′ UTR splice junctions, but not 3′ UTR length, to be a major determinant of NMD-sensitivity (86). When the presence of 3′ UTR splice junctions are accounted for, there is little or no difference in 3′ UTR length between NMD-sensitive and NMD-insensitive transcripts (84,87). Interestingly, other work has shown that in humans long 3′ UTR transcripts are protected from NMD by certain *cis*-sequence elements (88) or by the binding of PTBP1 near the stop codon (89), suggesting that many transcripts with naturally long 3′ UTRs are protected from NMD by RNA-binding proteins, explaining why some studies find individual transcripts with long 3′ UTRs to be NMD-sensitive but globally, most are likely protected. Our work suggests that the NMD pathway of moss is similar to that of mammals, and points to the likelihood that a similar mechanism already operated in the last eukaryotic common ancestor, although it remains to be seen whether the EJC itself is involved in NMD in moss, as in other plants and animals (12,17,90,91). The moss genome does encode two copies of all four of the core EJC components (Supplemental Table S9), but EJC-independent NMD involving splicing has also been observed in other species (84,85).

### PTC-containing isoforms with retained introns are largely resistant to NMD in moss

Retention of introns frequently introduces PTCs, and retained intron transcripts have been identified as NMD targets in many species (48,70,92). Retained intron transcripts have also been predicted to be NMD targets in plants (93). Recent work in *A. thaliana* and mammals have identified that many retained intron isoforms are resistant to NMD (22,23,71) and this is potentially due to nuclear localization of retained intron isoforms (71,94). We found this to be the case in moss, as retained intron transcripts are underrepresented amongst NMD targets, in contrast to other products of alternative splicing (Figures 5 and 6). However, a handful of retained intron transcripts are found in association with translating ribosomes in plants (95–97), suggesting that some escape the nucleus. Further work is needed to understand the details of nuclear localization of retained intron transcripts and whether such transcripts become subject to NMD if they escape the nucleus.

### NMD targets pose a problem for accurate annotation of genomes

Almost every day new genomes and transcriptomes are added to the public repositories and there has been tremendous progress in the bioinformatic prediction of transcripts in the recent years, however, the accurate reconstruction of spliced transcripts and prediction of their encoded ORFs still poses a difficult task. Our work here, and the work of others (23,98,99), demonstrate how prevalent AS-NMD is, and how it can cause problems for genome annotation approaches. Our data show that 3′ UTR splicing is a powerful predictor of NMD in moss, consistent with studies of other plants (12,17,23) and animals (14,86). Often, the longest ORF of a transcript is chosen when annotating a genome (100–102); however, when annotating NMD targeted transcripts, a stop codon introduced through splicing can be so early in the transcript that a the longest ORF starts from an internal methionine. Selection of the internal methionine masks this transcript as an NMD targeted transcript and simply appears to be a N-terminal truncation. Therefore, start codon selection should be influenced by what are likely translated AUGs, rather than the one leading to the longest ORF. Once an authentic start codon is selected, 3′ UTR splicing is likely to represent NMD targets or an artefact, such as a gene model fusion.

Additionally, not all transcripts with splice junctions in the 3′ UTR will be NMD targets. For example, many retained intron transcripts escape NMD due to nuclear detention (71,94). Without experimental validation of each transcript, computational annotations will need to classify transcripts with caution. Taking a more holistic approach to genome annotation, by taking into account the authentic start codon and type of splicing occurring in the transcript, should provide the research community a more accurate and useful resource.

### A biological rationale for SMG1

Notwithstanding the significant progress made in studying plant NMD using the *A. thaliana* model system, its exceptional status as one of only two known land plant species to lack the NMD kinase SMG1, limits its utility (29,30). The *SMG1* gene has been independently lost multiple times over the course of eukaryotic evolution, including in many fungal species and at least twice in land plants (29,30). Furthermore, the loss of SMG1 appears to have little to no effect on the NMD pathways in fruit flies and zebrafish (103–105). Therefore, in many organisms, SMG1 does not play a role in NMD at all. It is likely that another mechanism is able to readily replace SMG1 in the role of UPF1 activation upon PTC recognition, and this mechanism might be as ancient as SMG1 itself (29,30). It has been suggested that NMD is not an active process, but instead a passive process stemming from lack of translating ribosomes on the 3′ end of the gene (106). Intron retention-containing transcripts being NMD-insensitive might be consistent with a translation-dependent passive mechanism, however, much data supports the role of an active NMD pathway, for example: (107,108).

Here we used moss, a basal plant model in which SMG1 plays a role in NMD, to demonstrate that loss of *SMG1* affects several quality control pathways (Figure 7). We confirm our previous finding that SMG1 is important for the RNA quality control pathway, NMD (29). However, we also find that SMG1 appears to be needed for the correct functioning of the UPR and the DNA repair pathways. SMG1 could potentially act to integrate quality control pathways for RNA, DNA and protein in plants.

## DATA AVAILABILITY

Program to Assemble Spliced Alignments (PASA) is available at https://pasapipeline.github.io/. edgeR is an R package available from Bioconductor (https://bioconductor.org/packages/release/bioc/html/edgeR.html). NOISeq is an R package available from Bioconductor (https://www.bioconductor.org/packages/release/bioc/html/NOISeq.html). DESeq is an R package available from Bioconductor (http://bioconductor.org/packages/release/bioc/html/DESeq.html). TopHat is available at http://ccb.jhu.edu/software/tophat/index.shtml. Cufflinks is available at http://cole-trapnell-lab.github.io/cufflinks/. Datasets and scripts used in analysis of this publication are available as a Zenodo archive (https://doi.org/10.5281/zenodo.826164). htseq-count is part of the Python package HTSeq available at https://pypi.python.org/pypi/HTSeq. topGO is an R package available from Bioconductor (http://bioconductor.org/packages/release/bioc/html/topGO.html). caret is an R package available from CRAN (https://cran.r-project.org/web/packages/caret/). RNA-seq datasets have been deposited at the NCBI’s Short Read Archive under the BioProject accession number: PRJNA417704. The *smg1* knockout moss plants are available from the International Moss Stock Center (www.moss-stock-center.org/), *smg1*-1: #40832 and *smg1*-2: #40833.

## Supplementary Material

Supplementary Data
