# Supplementary material for: The loss of SMG1 causes defects in quality control pathways in Physcomitrella patens
Source: Nucleic Acids Res. 2018 Mar 27;46(11):5822–36. doi: 10.1093/nar/gky225 (PMC6009662; doi:10.1093/nar/gky225)
Supplement: Supplementary Data [file gky225_supplemental_files.zip › Supplemental_FigureS3.pdf]

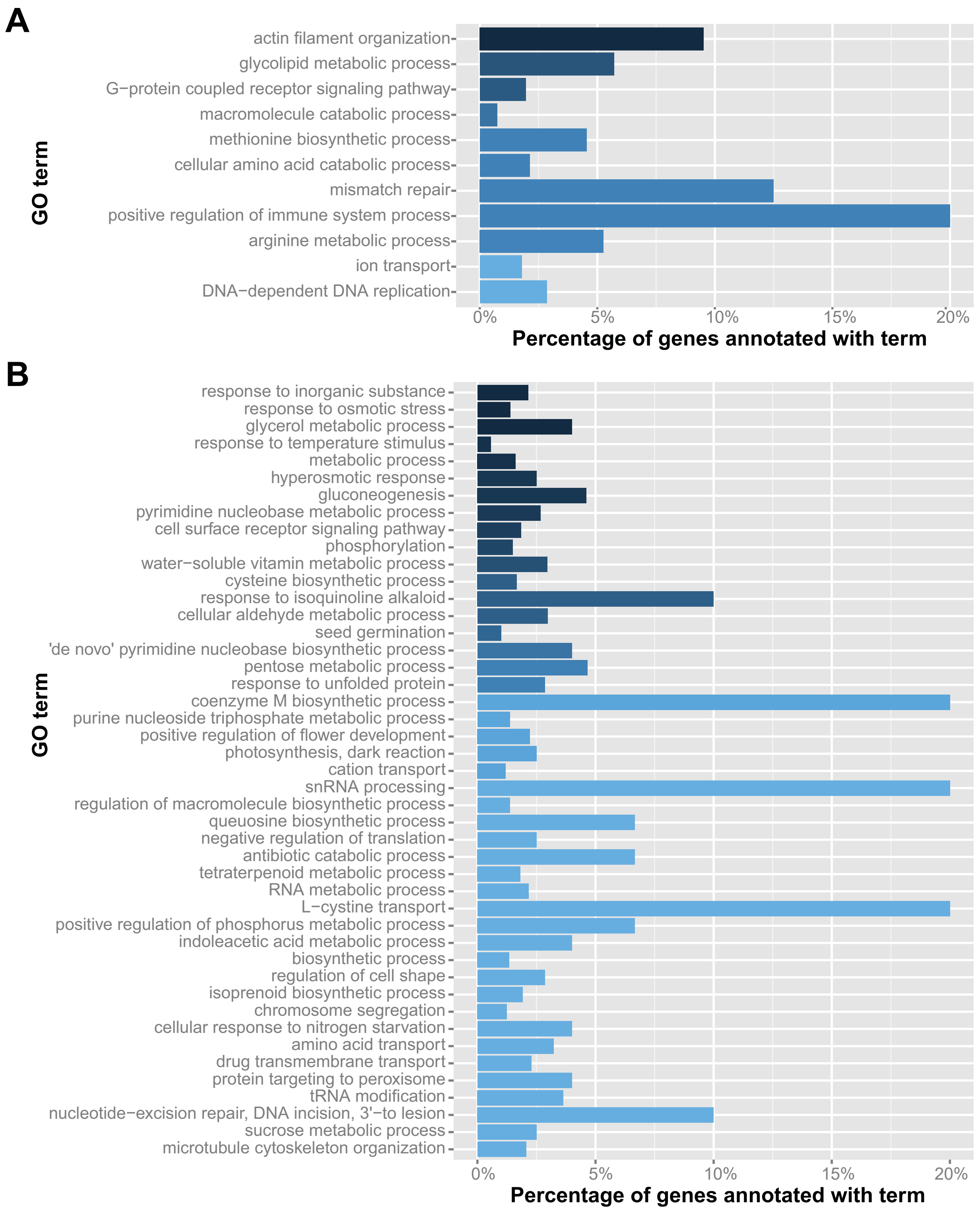


**Supplemental Figure S3.** Putative NMD targets span a range of diverse biological processes. (**A**) The sure set of AS-NMD target GO term enrichment. (**B**) The broad set of AS-NMD target GO term enrichment. Significantly over-represented GO biological process terms among the predicted AS-NMD targets (95% confidence). Terms are colored and sorted ascendingly according to *p*-value.
