# Supplementary material for: The loss of SMG1 causes defects in quality control pathways in Physcomitrella patens
Source: Nucleic Acids Res. 2018 Mar 27;46(11):5822–36. doi: 10.1093/nar/gky225 (PMC6009662; doi:10.1093/nar/gky225)
Supplement: Supplementary Data [file gky225_supplemental_files.zip › Supplemental_Figure_S1.pdf]

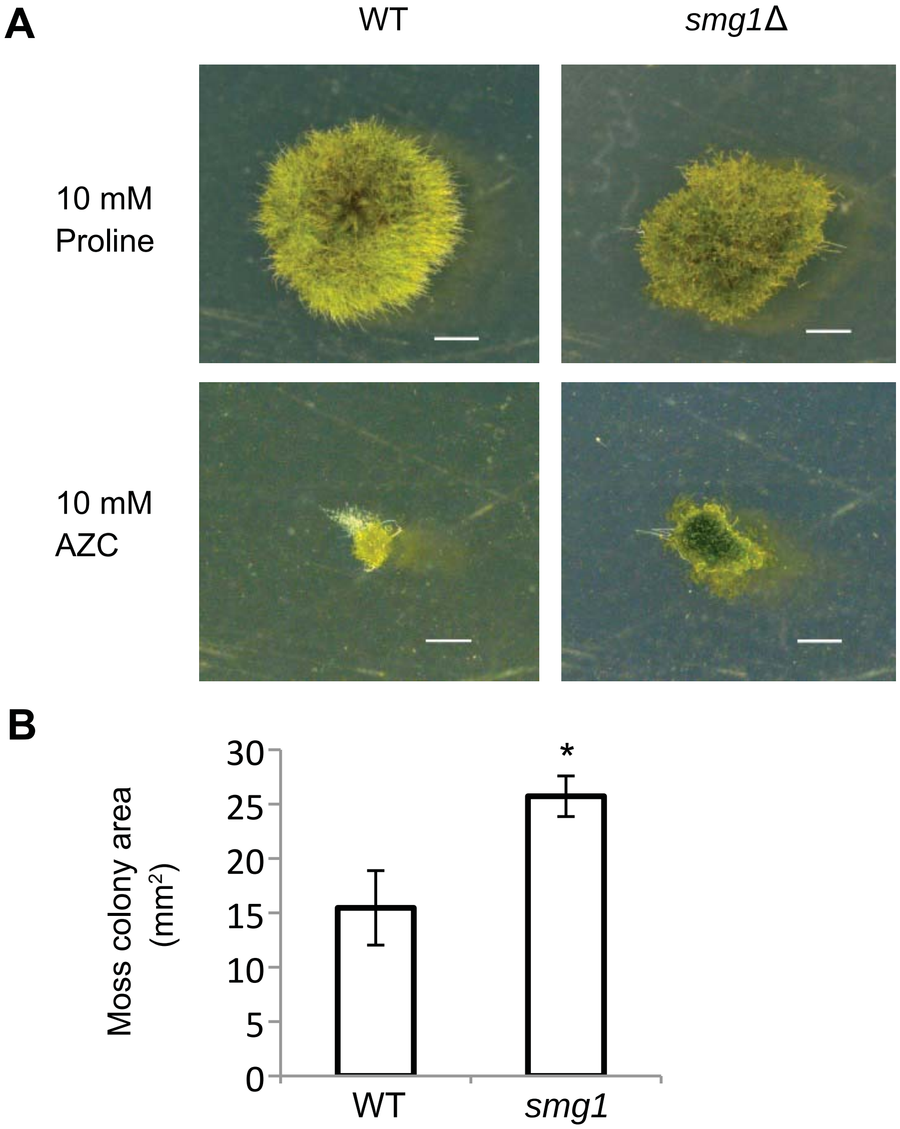

Supplemental Figure S1. *smg1* plants are partially resistant to the unfolded protein inducing drug AZC. (**A**) Three week old plants grown on 10 mM AZC or proline (control). Scale bar is 1 mm. (**B**) Moss colony size on 10 mM AZC or proline (control). n = 6-12. Asterisks indicate conditions with a statistically significant difference from WT (DMSO solvent control) using an unpaired t test (*$p < 0.05$; **$p < 0.01$; ***$p < 0.001$; ****$p < 0.0001$).
