# Supplementary material for: The loss of SMG1 causes defects in quality control pathways in Physcomitrella patens
Source: Nucleic Acids Res. 2018 Mar 27;46(11):5822–36. doi: 10.1093/nar/gky225 (PMC6009662; doi:10.1093/nar/gky225)
Supplement: Supplementary Data [file gky225_supplemental_files.zip › Supplemental_Figure_S2.pdf]

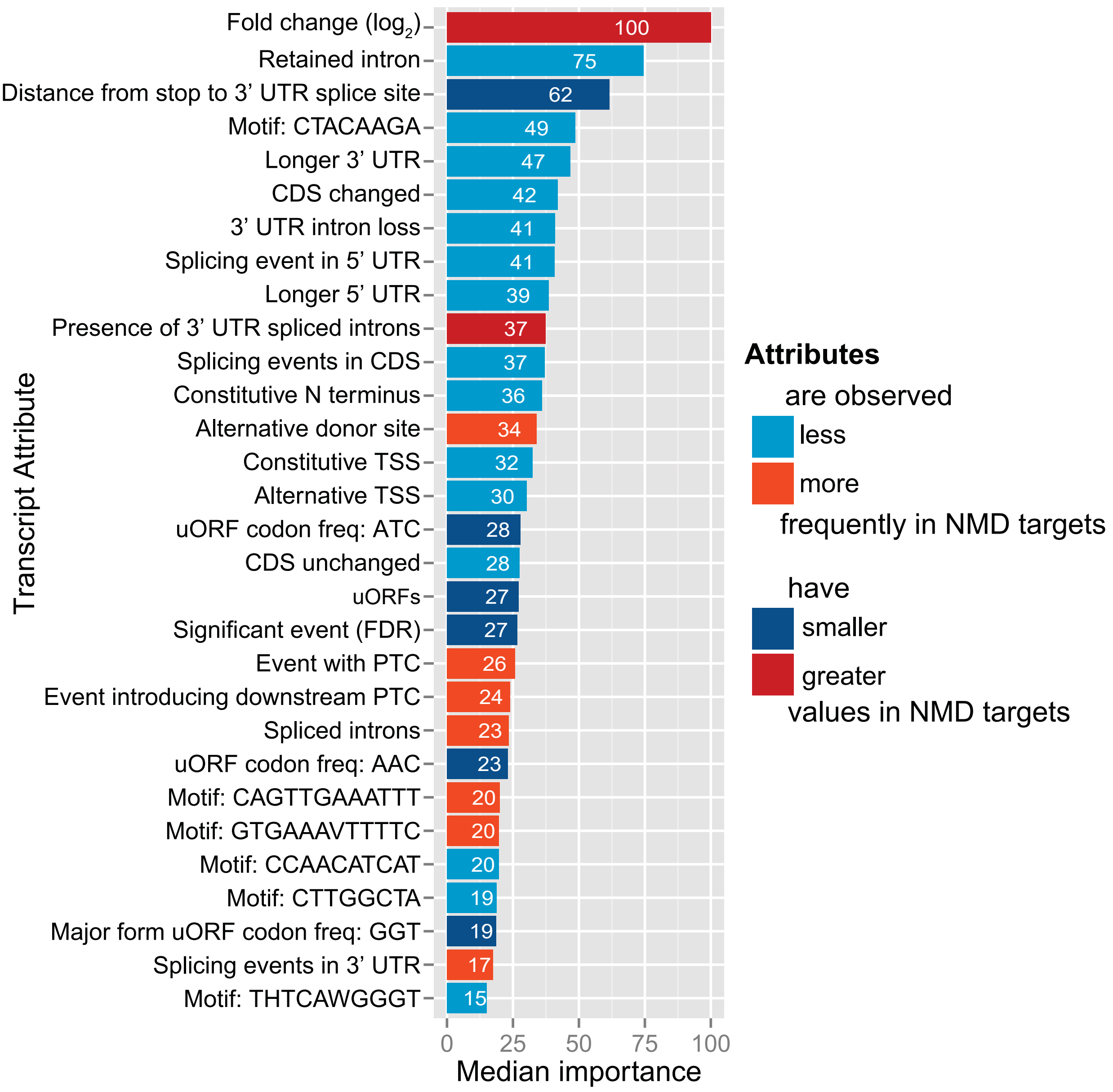

Supplemental Figure S2. Factors that influenced NMD target status in the machine learning approach. The top 30 transcript attributes that act as either positive (red or orange) or negative (light or dark blue) predictors of NMD-targeted status by the multiple machine learning approaches are shown. PTC was defined as a stop codon earlier than in the reference transcript. Transcript attributes are ranked by "median importance": Importance is the relative predictive power of each transcript attribute in identifying NMD-targeted events/transcripts. The median importance is taking from across the various machine learning tools used in this study.
