# Supplementary material for: The loss of SMG1 causes defects in quality control pathways in Physcomitrella patens
Source: Nucleic Acids Res. 2018 Mar 27;46(11):5822–36. doi: 10.1093/nar/gky225 (PMC6009662; doi:10.1093/nar/gky225)
Supplement: Supplementary Data [file gky225_supplemental_files.zip › Supplemental_Figure_S4.pdf]

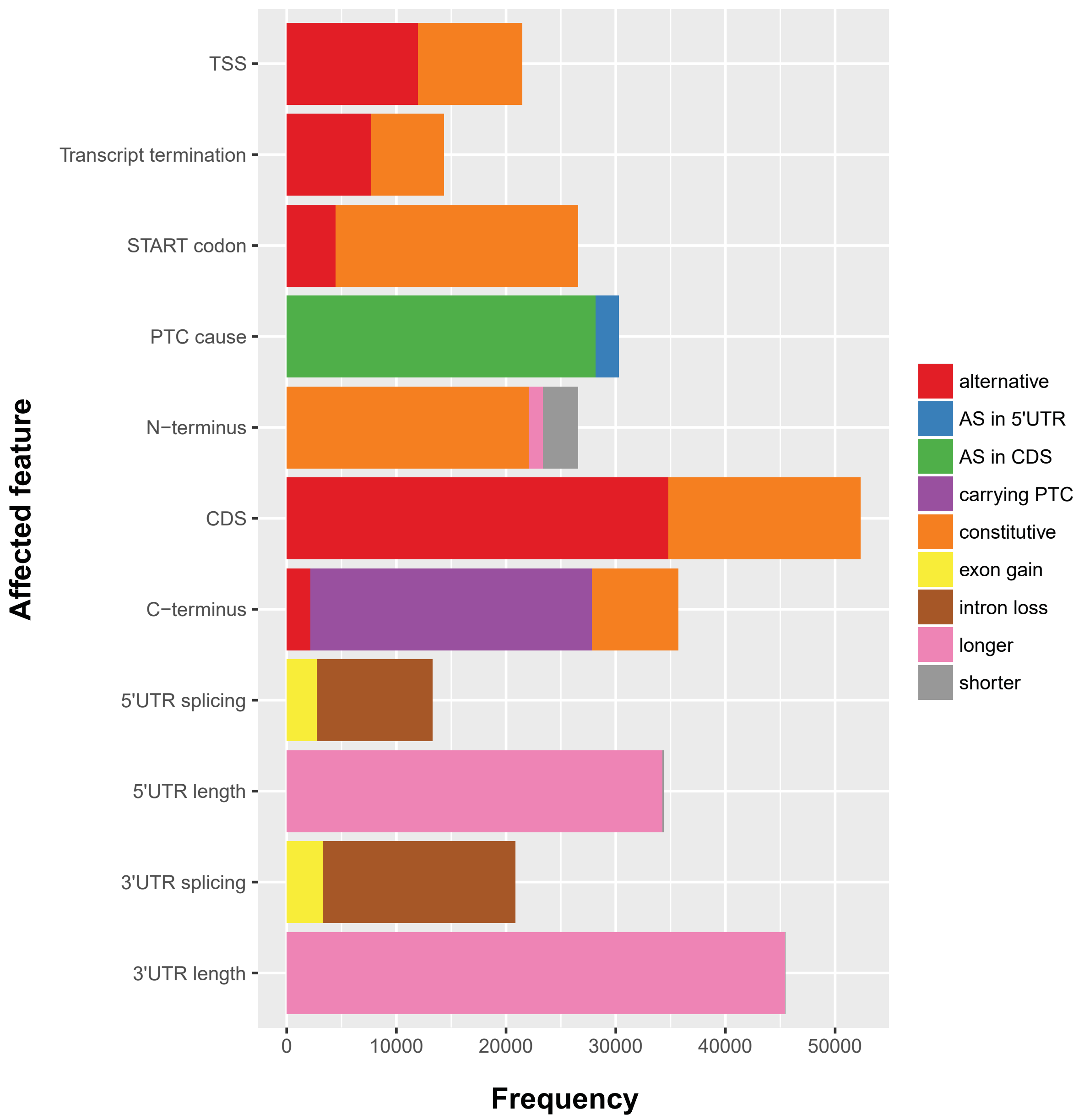

Supplemental Figure S4. Structural and functional consequences of alternative splicing in *P. patens*. Classification of location and structural and functional effects of AS events on gene structure and the mature transcripts' open reading frame, depicted are frequencies of classified events.
