# Supplementary material for: The loss of SMG1 causes defects in quality control pathways in Physcomitrella patens
Source: Nucleic Acids Res. 2018 Mar 27;46(11):5822–36. doi: 10.1093/nar/gky225 (PMC6009662; doi:10.1093/nar/gky225)
Supplement: Supplementary Data [file gky225_supplemental_files.zip › Supplemental_Table_list_and_Methods.pdf]

Supplemental Tables

Supplemental Table S1. List of primers used in this study.
Supplemental Table S2. Manually curated list of NMD targeted and non-NMD targeted splicing events as training set for the machine learning approach procedure.
Supplemental Table S3. Mapping rates of RNA-seq reads.
Supplemental Table S4. Differential gene expression analysis output.
Supplemental Table S5. Gene Ontologies (GO) terms associated with differentially expressed genes.
Supplemental Table S6. MapMan terms associated with differentially expressed genes.
Supplemental Table S7. Manually curated differentially expressed genes associated with the unfolded protein response.
Supplemental Table S8. Output from machine learning approach and AS-NMD analysis.
Supplemental Table S9. List of putative core exon-junction components in moss.

Supplemental Methods

Determining the main ORF of assembled transcripts

All open reading frames of the resulting transcript isoforms were screened for sequence homology using blastp against the NCBI nr protein database and a custom database comprising 20 published plant genomes (Zimmer et al., 2013) and the PFAM-A protein domain database utilizing the hmmsearch algorithm from the HMMER3 package. By ranking isoform/CDS combinations based on a) resulting homology evidences, b) protein length and c) isoform expression level (RPKM) the evolutionarily conserved, major i.e. reference isoform was chosen for each locus.

Identification of alternative splicing type and splicing consequence

Utilizing the alternative splicing inference module of PASA, splicing events at each locus were compared to the respective site in the reference isoform to determine their status being either constitutive or alternative. Based on the comparison with the reference CDS the custom algorithm also inferred the functional consequence of a splicing event by classification into 23 categories. The resulting gene models, inferred splicing events and functional consequence were integrated into the cosmoss.org genome browser as three separate and color-coded tracks (https://www.cosmoss.org/fgb2/gbrowse/physcome/; red=reference isoform, green=constitutive, orange alternative).

Identification of NMD targeting features and prediction of NMD targets using Ensemble (Machine) Learning

In order to utilize the resulting 76,329 classified splice events harbored within 32,499 transcript isoforms to identify common NMD targeting features, and to discriminate between true NMD targets and (secondarily) deregulated isoforms in the smg1 mutants, we collected 361 additional structural and sequence features in addition to the expression data described above. These features comprise both characteristics describing transcript isoforms (184 features) as well as the contained splicing events (177 features). First, the categorical values described earlier, the 10 AS types and the 23 functional consequence classes, were split up into binary features (e.g. AS-type.alt_acceptor =1|0). In addition, we collected numeric attributes describing the expression level in the mutants/wild type, measuring 46 features of gene structure (intron, exon, CDS, 5'UTR, 3'UTR) and uORFs, 207 sequence compositional features of transcript- and event-level contexts, 3 features describing cytosine methylation levels at splice sites as well as 67 frequencies of overrepresented motif and K-mers. Subsequently we compacted the feature vector by filtering near-zero variance and reducing correlated (>75%) features using the caret package. We generated two data sets: 1) comprising all filtered attributes including the expression data (EXP) and 2) comprising only structural and sequence features (NOEXP).

The following classifiers were selected: AdaBag, AdaBoost.M1, bstTree, C5.0Rules, cforest, ctree2, evtree, glmnet, pam, partDSA, pcaNNet, ranger, rf, rocc, rpart, rpart2, rpartCost, RRF, RRFglobal, svmLinear, svmLinear2, svmPoly and treebag. Each model results in an event is.NMD= TRUE|FALSE decision. All decisions were combined in a prediction ratio (the fraction of TRUE votes) for EXP (exp.pred.ratio) and NOEXP (noexp.pred.ratio) models separately and for all models combined (total.pred.ratio). Based on this data we inferred four rules for predicting NMD targeting potential of each detected splice site at different confidence levels:

1) **is.NMD.exp=TRUE** if exp.pred.ratio>=95% → 18.05 of 19 EXP models agree
2) **is.NMD.noexp=TRUE** if noexp.pred.ratio ==100% → 11 of 11 NOEXP models agree
3) **is.NMD.broad=TRUE** if (is.NMD.exp == TRUE or is.NMD.noexp==TRUE)
4) **is.NMD.sure=TRUE** if total.pred.ratio>=95% → at least 28.5 of 30 models say conclude is.NMD=TRUE

Decisions based on the rules 3) and 4), provide different levels of confidence in the prediction whether a splicing event is leading to NMD or not. Predictions based on rule 4) are very stringent/sure and should minimize false positives (training set performance: sensitivity 100% and specificity 100%), while missing out more obscure true targets which e.g. are not expressed under the conditions monitored in the mutant and thus can only be identified by employing a broader definition (e.g. relying solely on structural/sequence features) implemented in rule 3) which likely contain a higher false positive rate (sensitivity: 100% and specificity: 82%).

Alternative splicing type nomenclature

From the PASA output, throughout the main text of the paper and the figures, “Retained exons” are renamed “Included exons”.
